# Patient with Disorders of Sex Development (DSD): A Case Report from a Tertiary Care Hospital in Thiruvananthapuram, India

**Published:** 2019

**Authors:** Pongillyathundiyil Sasidharan Sreejith, Sheila Balakrishnan, Vaikom Hariharan Sankar, Remya Syamala, Reji Mohan, Sankar Sundaram, Krishna Govindan, Kaleeluvilayil Raghavan Nair Chandramohanan Nair

**Affiliations:** 1- Multi Disciplinary Research Unit, Government Medical College, Thiruvananthapuram, India; 2- Department of Reproductive Medicine and Surgery, Sree Avittom Thirunal Hospital, Government Medical College, Thiruvananthapuram, India; 3- Department of Pediatrics, Sree Avittom Thirunal Hospital, Government Medical College, Thiruvananthapuram, India; 4- Child Development Centre, Government Medical College, Thiruvananthapuram, India; 5- Department of Pathology, Government Medical College, Kottayam, India; 6- Department of Anatomy, Government Medical College, Thiruvananthapuram, India

**Keywords:** 46 XX male syndrome, Azoospermia, SRY gene, Y chromosome microdeletion

## Abstract

**Background:**

46 XX male syndrome, a rare case of infertility was first reported by de la Chapelle in 1964. In newborn males, the incidence rate of the syndrome varies from 1/9000 to 1/20000. Here, a case of 46 XX male syndrome is reported with clinical, biochemical and genetic changes of the patient and normal masculine features.

**Case Presentation:**

A 29 year old male with infertility registered at the Sree Avittom Thirunal Hospital of Government Medical College, Thiruvananthapuram for fertility treatment. He was diagnosed with non obstructive azoospermia in repeated semen analysis. Chromosomal analysis on peripheral blood lymphocytes has revealed 46 XX male syndrome and the result was confirmed with Fluorescent In situ Hybridization (FISH). Real time polymerase chain reaction failed to detect genes on azoospermia factor regions, AZFa, AZFb and AZFc of Y chromosome, but detected SRY gene positivity. Masculine features of patient were normal except small sized testis, ejaculatory dysfunction and azoospermia.

**Conclusion:**

Appearance of the external genitalia will be generally normal in 46 XX with SRY positive males and generally difficult to identify before puberty because there will not be any significant clinical indication. The present case report demonstrates that mere physical or clinical examination may not disclose the genetic defects. Therefore, in addition to general examination, it is essential to perform genetic analysis on men with infertility.

## Introduction

In 1964, a rare case of male infertility of 46 XX male syndrome was first reported by de la Chapelle (1). In newborn males, the incidence rate of the syndrome varies from 1/9000 to 1/20000 (2, 3). The International Consensus Conference on the Management of Intersexuality held in October 2005 accepted the new nomenclature for XX male syndrome to 46 XX testicular Disorders of Sex Development (DSD). The major reason for 46 XX male condition is the un-equal crossing over or interchange of a fragment of the short arm of the Y chromosome containing SRY gene with the X chromosome during paternal meiosis (4–8). Majority of 46 XX males show normal phenotype, but 10–15% exhibit diverse clinical features (9, 10). The present study explains the structural and numerical abnormalities of chromosomes along with clinical and biochemical parameters of the patient.

## Case Presentation

A 29 year old male visited the fertility center of Sree Avittom Thirunal Hospital of Government Medical College, Thiruvananthapuram for fertility treatment. Consent was taken from the patient before the study. The present finding of unexpected association between DSD and infertility in Kerala population was observed on 1st March 2018 at Sree Avittom Thirunal Hospital of Government Medical College, Thiruvananthapuram, Kerala, India during karyotyping and molecular characterization of azoospermic male patients.

Physical examination and medical history showed that the patient having height of 170 *cm* and weight of 75 *Kg* had no history of smoking, alcohol consumption and tobacco chewing. He has been using modern electronic gadget (Mobile phone) for the last five years and did not have any history of orchitis, STDs, psychological and endocrine disorders. Clinical findings revealed that the subject did not have palpation of testis, cryptorchidism, testicular torsion and changes in his extremities. Family history of patient did not show any infertility related problems. The study subject showed normal male secondary sex characters, penile length and androgenic hair pattern and no erection problem, gynecomastia and hypospadias were observed. The testis volume was <2 *ml* and both testicles were soft and atrophic. Major noticeable changes observed during clinical evaluation were small sized testis and ejaculatory dysfunction. Semen analysis was done on sample collected after 3 days of abstinence and the results confirmed non obstructive azoospermia on repeated analysis. Volume was 2 *ml*, pH=7.4, liquefaction time 15 *min*, WBC 0–1 *pc/ml* and fructose was present. Percutaneous Epididymal Sperm Aspiration (PESA) was done on both sides and sperm could not be retrieved. The couples opted for artificial insemination by donor (AID). Ultrasonography (USG) of scrotum showed right testis to be 1.2 *cm* × 1.8 *cm* × 1.4 *cm* in size with normal echotexture. Epididymis was normal with dimensions of 1 *cm* × 0.7 *cm*. Left testis measured 1.3 *cm* × 1.7 *cm* × 0.9 *cm* in size with no indication of varicocele. Transrectal ultrasound (TRUS) showed prostate with 38 *mm* X 35 *mm* × 20 *mm* size. No lesions were observed in any of the transrectal components. Urinary bladder was normal with no dilation in the seminal vesicles and ejaculatory ducts. Hormonal analysis showed an increase in Follicle Stimulating Hormone (FSH) to 35 *mIU/ml* (1–12 *mIU/ml*) and Luteinizing Hormone (LH) to 19.9 *mIU/ml* (1.5–10 *mIU/ml*) whereas testosterone to 3.2 *ng/ml* (2.45–10 *ng/ml*) and prolactin to 6.7 *ng/ml* (3.1–16.5 *ng/ml*) which were within the limit of reference range.

**Figure 1.**
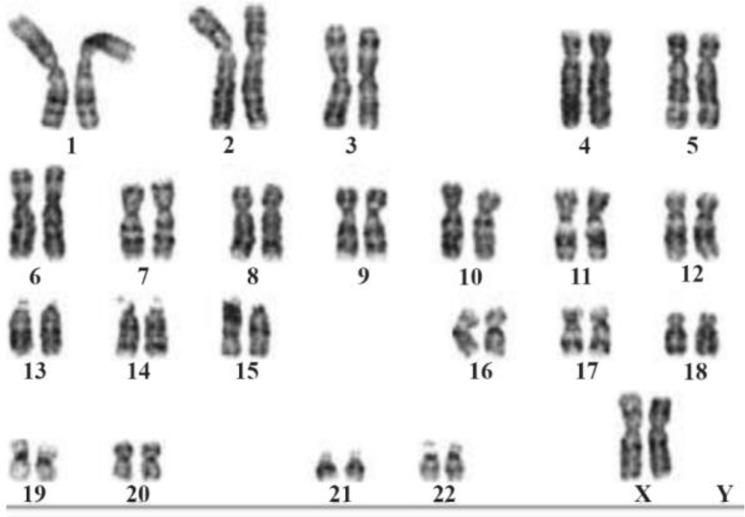
The Karyotype shows the patient is having two X chromosome and no Y chromosome

Chromosomal analyses of peripheral lymphocytes were used for identifying the numerical and structural aberrations of chromosomes. GTG banding was done for karyotyping. A total of 30 metaphase chromosomes were analyzed and the results showed that the patient had 46 XX karyotype (Figure 1) known as de la Chapelle syndrome with no evidence of other structural/numerical chromosomal abnormalities including mosaicism.

**Figure 2.**
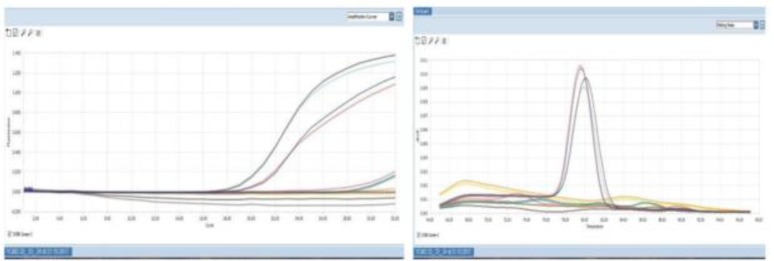
Real time PCR shows both amplification curve and Melt peak. No amplification of AZFa, AZFb and AZFc in test sample, but SRY and ZFX, the internal control genes show amplification

Molecular analysis of the Y-chromosome specific STS region by real time PCR and Uniplex PCR amplification of Y chromosome specific six sequence tagged sites (STS) primers were used for identifying AZF gene microdeletion (Table 1). The primers were designed based on EMQN guidelines (11). Primers against SRY gene and a unique fragment in X chromosomes and ZFX gene were used as internal controls. Real time PCR analysis showed absence of entire AZFa, AZFb and AZFc region but the presence of SRY gene was confirmed as shown in figure 2. All STS primers were amplified in the male positive control. The female genomic DNA failed to amplify in any of the STS primers and the SRY gene, however, showed amplification of the ZFX gene. These findings confirmed that the patient is 46 XX male with the presence of SRY gene.

**Table 1.** Primers used for Real Time PCR work

**No.**	**Primer**	**Forward 5′-3′**	**Reverse 5′-3′**
**1**	SRY	TATCCTGGACGTTGCCTTTAC	GAGAAGCTCTTCCTTCCTTTG
**2**	ZFX	GTTATTGAATCGCCACCTC	ACCCTTACCACACTCCACAC
**3**	SY84	GCTGGAGATTCAGTGGGAC	TGGAGGCTTCATCAGCAAG
**4**	SY86	GATGTCAAGGCTGCAGATC	GCCCAGTCTTTGGGATTTC
**5**	SY127	CTTATATGGGTGAGCCAGATG	CACAGACAGGGAAATCTCCAG
**6**	SY134	GCATTCTGTGACTGAAGAGG	AACGTGGGAACCTCACAAG
**7**	SY254	GGTGGAATTGATGCTAGGG	TTATCCCCGAATGACCAGC
**8**	SY255	GCTGAGTTACAGGATTCGG	TGGTAGTTAATCCTCCTCCTCC

**Figure 3.**
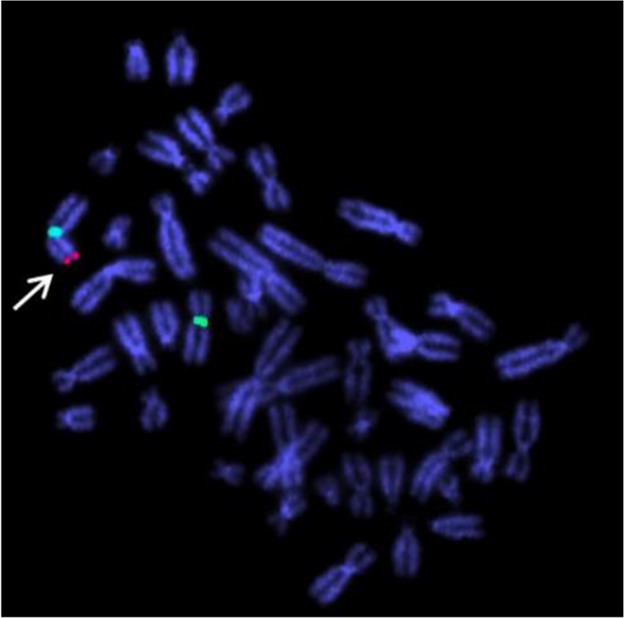
Dual color FISH data shows presence of two X chromosomes and SRY gene on one of the X chromosome

Fluorescent in situ hybridization (FISH) analysis was performed on metaphase chromosomes from cultured peripheral lymphocytes to confirm the 46 XX male condition using LSI SRY/CEPX probe (Vysis Inc., USA) localized on Yp11.3 and p11.1-q11.1 of X chromosome. The probe hybridization showed two signals for CEPX (Green) at the centromeric region of both the X chromosome and one signal for SRY (Red) on terminal region of one X chromosome in all the metaphases analyzed (Figure 3). This finding confirmed the 46 XX karyotype, a case of DSD.

## Discussion

The XX male syndrome is a rare genetic disorder. Its phenotype may vary and range from a severe impairment of the external genitalia to a normal male phenotype with infertility. Appearance of external genitalia is normal in 46 XX SRY positive males and difficult to identify before puberty because of lack of any significant clinical indication except undescended small testis. This syndrome accounts for 2% of cases of male infertility. Most of the time, the defect is diagnosed only during molecular investigations associated with hypogonadism, gynecomastia or infertility whereas, 46 XX SRY negative males can be identified immediately after birth owing to inadequate virilization of external genital organs. Early and accurate diagnosis of DSD is very important in order to prevent post-surgical, social, psychological and sexual complications.

This case study presents the clinical, hormonal and molecular data of an individual with XX male syndrome. The patient was phenotypically male with no ovaries, uterus, cervix, vagina and other female genitalia. This study also emphasizes the value of karyotyping and molecular diagnosis of patients having disorders of sex development, as phenotype does not always correlate with genotype. The identification of such cases is multi-directional involving thorough physical examination, proper clinical investigation and molecular cytogenetic testing to arrive at a precise conclusion. Males with testicular DSD can be divided into three categories of typical group characterized by male phenotype, ambiguous genital group and the real hermaphrodites (12). In 90% of patients of the first category, SRY gene exists on the short arm of X chromosome and it is the vital gene responsible for testicle development normally found on Y chromosome. Like previous studies (1–5, 7, 12), the present finding demonstrates a phenotypically male but genetically female case.

## Conclusion

General clinical examination may not be sufficient for identification of 46 XX DSD. A detailed hormonal and molecular analysis is essential for detecting genetic defects. This study emphasizes to perform molecular analysis on men with infertility to reveal genetic defects in addition to general examination.
